# Drive-By Bridge Damage Identification Using Successive Variational Modal Decomposition and Vehicle Acceleration Response

**DOI:** 10.3390/s25123752

**Published:** 2025-06-16

**Authors:** Xiaobiao Jiang, Kun Ma, Jiaquan Wu, Zhengchun Li

**Affiliations:** 1 Faculty of Civil Engineering and Mechanics, Kunming University of Science and Technology, Kunming 650500, China; 2 Faculty of Science, Kunming University of Science and Technology, Kunming 650500, China

**Keywords:** bridge damage identification, drive-by method, successive variational mode decomposition (SVMD), structural health monitoring

## Abstract

Using a two-axle test vehicle, a new drive-by-based bridge damage identification method is proposed in this study. The method firstly obtains the vehicle acceleration response of a vehicle passing through an undamaged bridge and a damaged bridge; then, the acceleration response is processed using successive variational modal decomposition (SVMD) to obtain the intrinsic modal function (IMF) corresponding to the driving frequency; finally, the difference of the IMF is used to construct a damage indicator for damage identification of the bridge. The main findings of this study are as follows: (1) the constructed damage index can successfully identify single and multiple damages of bridges; (2) even in the case of pavement roughness, the proposed damage index is still able to identify the location of the damage; (3) the constructed damage index is not only applicable to simply supported bridges, but also applicable to the damage identification of continuous bridges; (4) the experiment shows that the proposed damage index can successfully identify the damage location, but the local vibration of the vehicle and the measurement noise interfere with the damage identification effect severely.

## 1. Introduction

Bridge damage often results in changes to modal parameters, such as natural frequency, mode shapes, and damping. The accuracy of structural vibration-based monitoring methods relies on precisely identifying these modal parameters [1]. Traditionally, this bridge health diagnosis (BHD) approach involves installing sensors directly on the bridge to capture data and monitor changes in its dynamic characteristics; this method is referred to as the direct method.

Due to the need for numerous sensors, the traditional direct method faces challenges such as installation difficulties, short equipment lifespan, low measurement accuracy, and high costs, making monitoring the vast number of small- to medium-span bridges impractical. To overcome these drawbacks, Yang et al. proposed the Vehicle Scanning Method (VSM), which involves installing a few sensors on a moving detection vehicle to capture vehicle vibration responses to identify bridge modal parameters [2]. VSM enhances the comprehensiveness of measurements and offers advantages such as no additional excitation required, uninterrupted traffic flow, and reusable equipment, thereby demonstrating high economic and practical value. Currently, VSM is primarily employed to inspect single-type bridges. However, its potential application for longer bridges is significant, suggesting that it could become an effective tool for the rapid assessment of regional bridge networks in the future [3,4,5].

In vibration-based structural health monitoring systems, signal processing techniques are central, encompassing applications such as data denoising, feature extraction, and damage identification. The Fast Fourier Transform (FFT), a fundamental method for converting time-domain signals into frequency-domain signals, is widely used in various fields, including structural vibration control and bridge engineering [6,7,8]. Wavelet Transform (WT) decomposes signals at multiple scales using a series of basis functions localized in frequency and time, making it particularly suitable for non-stationary signals and signal denoising [9,10]. Lorenzo et al. [11] proposed a damage detection method that combines Continuous Wavelet Transform (CWT) with a sparse autoencoder, utilizing the vertical acceleration of the leading bogie of trains to assess bridge health. The study demonstrated promising damage detection performance, even under the presence of measurement noise and inaccuracies in speed estimation, particularly when a batch of 40 trains was considered. Empirical Mode Decomposition (EMD), known for its strong adaptability and lack of need for predefined basis functions, has been widely applied in bridge engineering [12,13,14]. Advanced signal processing methods such as Variational Mode Decomposition (VMD) and Ensemble Empirical Mode Decomposition (EEMD) with adaptive noise have further demonstrated significant value in bridge engineering applications [15,16,17,18,19]. Selecting an appropriate and adaptive method for bridge health monitoring is crucial, and techniques such as Bayesian inference and machine learning provide potent tools for uncertainty quantification, damage identification, and predictive analysis [20,21].

To enhance the bridge modal identification capability of the Vehicle Scanning Method (VSM), data processing and filtering techniques are crucial. Although previous studies have primarily utilized Empirical Mode Decomposition (EMD), its issues with modal aliasing and extended computation time have limited its applicability [22,23,24,25]. Consequently, scholars have proposed Variational Mode Decomposition (VMD) for bridge health monitoring, which addresses the modal aliasing problem inherent in EMD and offers orthogonal modal components with well-defined frequencies [26,27,28,29]. However, the sensitivity of VMD to the pre-set number of modes and central frequencies and parameter selection can affect the robustness of the decomposition results. Although VMD has been widely used, its performance is highly dependent on manually pre-defined parameters, such as the number of modes and the bandwidth constraint. In contrast, Successive Variational Mode Decomposition (SVMD) adaptively determines the number of modes by sequentially decomposing the signal and selecting the optimal center frequency for each mode. This approach avoids redundant mode extraction, reduces computational cost, accelerates convergence, and is thus more suitable for real-time applications [30]. This study introduces SVMD for processing VSM data to address these concerns. SVMD excels in the accuracy of signal decomposition and handling complex non-stationary signals, and it can adaptively adjust parameters, thereby enhancing the system’s performance in modal and damage identification [31,32,33,34].

Compared to methods such as Empirical Mode Decomposition (EMD) and Ensemble Empirical Mode Decomposition (EEMD), Variational Mode Decomposition (VMD) offers higher time–frequency resolution and stronger noise robustness, enabling more effective extraction of modal features from non-stationary signals. However, its performance is highly dependent on manually pre-defined parameters, including the number of modes and the bandwidth constraint for each mode. To address these limitations, Nazari and Sakhaei [35] proposed a novel approach—Successive Variational Mode Decomposition (SVMD)—which extracts signal components sequentially. SVMD demonstrates superior decomposition capability, particularly when the number of intrinsic components is unknown. Its application in bridge structural monitoring is still in the early stages. In this study, we, for the first time, integrate SVMD with a two-axle vehicle model for bridge damage identification, highlighting its methodological novelty and potential for advanced structural health monitoring. In this study, a commonly used Band-Pass Filter (BPF) is employed as a preprocessing step. The BPF is first applied to eliminate frequency components outside the expected spectral range. Subsequently, the filtered vibration signals are decomposed using the Successive Variational Mode Decomposition (SVMD) method. According to [36], the frequency band corresponding to the driving frequency contains dense multi-order bridge information and exhibits high sensitivity to stiffness variations. Therefore, the Intrinsic Mode Function (IMF) associated with the driving frequency is extracted and utilized for identifying potential bridge damage.

The organization of this paper is as follows: Section 2 introduces the coupling principles between a two-axle and the bridge. Section 3 briefly introduces the SVMD technique used for signal processing. Section 4 verifies the feasibility of the damage identification index through numerical simulation. Section 5 investigates the effect of various parameters such as vehicle speed, pavement roughness, and bridge span. Section 6 conduct experimental research. Section 7 presents the conclusions.

## 2. Vehicle-Bridge Coupling Analysis

Previous VSM studies have predominantly relied on a single-axle vehicle model—such as mass-spring systems or quarter-car models. However, this model lacks self-balancing capabilities and propulsion, requiring an additional towing vehicle, which increases complexity and cost. In contrast, limited research has been conducted on more commonly used two-axle vehicles. This study introduces a two-axle vehicle model, which offers greater interaction with the bridge surface through multiple contact points. As a result, it can capture richer vibration response data from the bridge, thereby enhancing the sensitivity to local damage. Therefore, studying the application of two-axle vehicles is particularly important.

**Figure 1:**
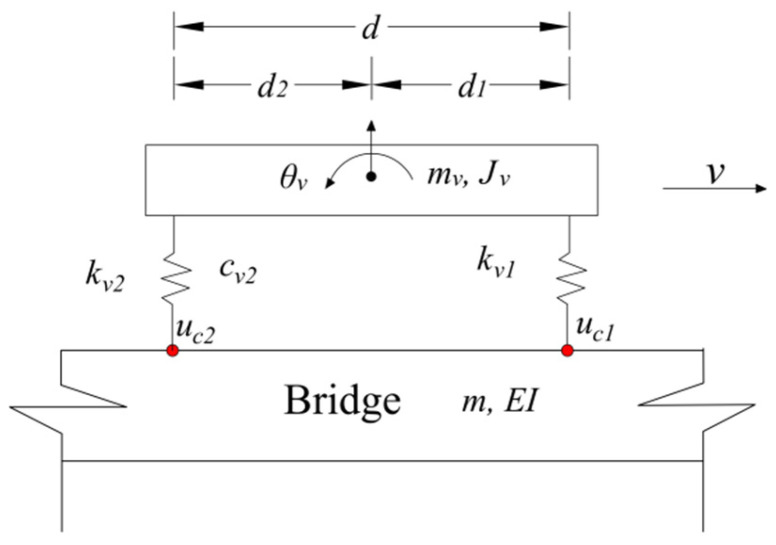
Two-axle vehicle–bridge coupling model.

The adopted two-axle vehicle model is shown in Figure 1. The vehicle travels at a constant speed *v* on a supported beam. The detection vehicle is modeled as a two-degree-of-freedom (DOF) system, with the vehicle body considered a rigid body with two DOFs: vertical translation *y_v_* and rotation θ. The moment of inertia of the vehicle is denoted as *J_v_*. The wheelbase between the front and rear axles is *d*, the distance from the vehicle’s center of gravity to the front axle is *d*_1_, and the distance to the rear axle is *d*_2_. The stiffness of the front and rear axles are denoted as *k_v_*_1_ and *k_v_*_2_, respectively. The simply supported beam is modeled as a Euler beam. The span length of the simply supported beam is *L*, the mass per unit length is *m*, and the flexural rigidity of the cross-section is *EI*. To simplify the theoretical derivation of the relationship between vehicle response and bridge vibration, the effects of road surface roughness were neglected in the analytical model. However, these factors were incorporated into the numerical simulations. This assumption is considered reasonable, as the inclusion of road roughness does not eliminate the bridge-induced vibration components present in the vehicle response. Instead, it introduces additional vibration components or affects the amplitude of the bridge-induced signals [37].

The bridge displacement can be written as [37]

(1) $$u\left(x,t\right)=\sum_{n=1}^{N}\sum_{k=1}^{2}\frac{\Delta_{st,n}}{2\left(1-S_{n}^{2}\right)}\left[\sin\frac{n\pi v\left(t-t_{k}\right)}{L}-S_{n}\sin\omega_{bn}\left(t-t_{k}\right)\right]\times \left[H\left(t-t_{k}\right)-H\left(t-t_{k}-\Delta t\right)\right]\times \sin\frac{n\pi x}{L}$$

where $\Delta_{st,n}$ is the static deformation of the *n* th mode caused by the load on the front or rear axle $p_{k}$, $t_{k}$ is the time when the front and rear axles enter the bridge, and the expressions for the parameters are as follows:

(2) $$\Delta_{stn}=-\frac{2m_{v}gL^{3}}{EIn^{4}\pi^{4}},S_{n}=\frac{n\pi v}{L\omega_{bn}},\;\omega_{bn}=\frac{n^{2}\pi^{2}}{L^{2}}\sqrt{\frac{EI}{m}},\;t_{k}=\frac{(k-1)d}{\nu }$$

In the equation, *t_k_* denotes the time when the k-th wheel enters the bridge. The equilibrium equations for the vertical translation are given by [37]

(3) $$\begin{array}{l} \ddot{y}=-\sum_{n=1}^{N}\frac{\omega_{y}^{3}\Delta_{st,n}\sin(\omega_{y}t)}{8(1-S_{n}^{2})}A_{n}+\sum_{n=1}^{N}\frac{\omega_{y}^{4}\Delta_{st,n}\cos(\omega_{y}t)}{8(1-S_{n}^{2})}B_{n}\\ +\sum_{n=1}^{N}\frac{\omega_{y}^{2}\Delta_{st,n}\omega_{n}^{2}}{8(\omega_{y}^{2}-\omega_{n}^{2})(1-S_{n}^{2})}\left[\cos(\omega_{n}t)+2\cos(\omega_{n}t-\omega_{d})+\cos(\omega_{n}t-2\omega_{d})\right]\\ +\sum_{n=1}^{N}\frac{\omega_{y}^{2}\omega_{bln}^{2}\Delta_{st,n}S_{n}}{8(\omega_{y}^{2}-\omega_{bln}^{2})(1-S_{n}^{2})}\left[\cos(\omega_{bln}t)+\cos(\omega_{bln}t+\omega_{d})+\cos(\omega_{bln}t-\omega_{bln})+\cos(\omega_{bln}t-\omega_{bln}+\omega_{d})\right]\\ -\sum_{n=1}^{N}\frac{\omega_{y}^{2}\omega_{brn}^{2}\Delta_{st,n}S_{n}}{8(\omega_{y}^{2}-\omega_{brn}^{2})(1-S_{n}^{2})}\left[\cos(\omega_{brn}t)+\cos(\omega_{brn}t-\omega_{d})+\cos(\omega_{brn}t-\omega_{bdn})+\cos(\omega_{brn}t-\omega_{bdn}-\omega_{d})\right] \end{array}$$

where the coefficients are defined as

(4) $$\omega_{y}=\sqrt{\frac{2k_{v}}{m_{v}}}$$

(5) $$\begin{array}{l} A_{n}=\frac{\omega_{n}\left[2\sin\left(\omega_{d}\right)+\sin\left(2\omega_{d}\right)\right]}{\left(\omega_{y}^{2}-\omega_{n}^{2}\right)}+\frac{\omega_{bln}S_{n}\left[\sin\left(\omega_{bdn}\right)-\sin\left(\omega_{d}\right)+\sin\left(\omega_{bdn}-\omega_{d}\right)\right]}{\left(\omega_{y}^{2}-\omega_{bln}^{2}\right)}\\ -\frac{\omega_{brn}S_{n}\left[\sin\left(\omega_{bdn}\right)+\sin\left(\omega_{d}\right)+\sin\left(\omega_{bdn}+\omega_{d}\right)\right]}{\left(\omega_{y}^{2}-\omega_{brn}^{2}\right)} \end{array}$$

(6) $$\begin{array}{l} B_{n}=\frac{2+2\cos\left(\omega_{d}\right)}{\omega_{y}^{2}}-\frac{1+2\cos\left(\omega_{d}\right)+\cos\left(2\omega_{d}\right)}{\left(\omega_{y}^{2}-\omega_{n}^{2}\right)}-\frac{S_{n}\left[1+\cos\left(\omega_{bdn}\right)+\cos\left(\omega_{d}\right)+\cos\left(\omega_{bdn}-\omega_{d}\right)\right]}{\left(\omega_{y}^{2}-\omega_{bln}^{2}\right)}\\ +\frac{S_{n}\left[1+\cos\left(\omega_{bdn}\right)+\cos\left(\omega_{d}\right)+\cos\left(\omega_{bdn}+\omega_{d}\right)\right]}{\left(\omega_{y}^{2}-\omega_{brn}^{2}\right)} \end{array}$$

where

(7) $$\omega_{d}=\frac{2n\pi d}{L},\omega_{n}=\frac{2n\pi v}{L},\omega_{bdn}=\frac{2d}{v}\omega_{bn},\omega_{bln}=\omega_{bn}-\frac{n\pi v}{L},\omega_{brn}=\omega_{bn}+\frac{n\pi v}{L}$$

## 3. Brief of SVMD Technique

Selecting an effective data processing method is crucial to identifying bridge damage. To enhance the accuracy of VSM in identifying bridge damage, this section will briefly introduce the principles of SVMD.

VMD is a commonly used signal decomposition method that decomposes the original data into K modal decomposition functions, known as Intrinsic Mode Functions (IMFs). This decomposition approach reduces the non-stationarity and non-linearity of the original data, thereby improving the accuracy of predictions. Selecting an appropriate number of modal decomposition functions is crucial in the VMD decomposition process. Choosing too few can result in high residual complexity, which affects prediction accuracy, while choosing too many can lead to over-decomposition. This study employs SVMD to decompose the measurement vehicle signals to address this issue. Unlike VMD, SVMD does not require a pre-set number of decomposition functions K and reduces computational complexity. The computational formula is as follows:

For a signal *f(t)*, it is decomposed into *L* modes and a residual signal:

(8) $$f(t)=u_{L}(t)+f_{r}(t)$$

In the Equation, the residual signal includes the unprocessed part $f_{r}(t)$ as well as the sum of the previously obtained modes $\sum_{i=1}^{L-1}u_{i}(t)$. The residual signal expression is

(9) $$f_{r}(t)=\sum_{i=1}^{L-1}u_{i}(t)+f_{u}(t)$$

The core idea of SVMD is to iteratively optimize and extract the best-fitting frequency and amplitude parameters within the current frequency range of the signal. This approximation and optimization process is implemented using the method of Lagrange multipliers. During the (n+1)th iteration, the modal component *u_L_* is updated using the following formula:

(10) $${\hat{u}}_{L}^{n+1}(\omega )=\frac{\hat{f}(\omega )+\alpha^{2}{\left(\omega -\omega_{L}^{n}\right)}^{4}{\hat{u}}_{L}^{n}(\omega )+\frac{\hat{\lambda }(\omega )}{2}}{\left[1+\alpha^{2}{\left(\omega -\omega_{L}^{n}\right)}^{4}\right]\left[1+2\alpha {\left(\omega -\omega_{L}^{n}\right)}^{2}+\sum_{i=1}^{L-1}\frac{1}{\alpha^{2}{\left(\omega -\omega_{i}\right)}^{4}}\right]}$$

In this formulation, α is the balancing parameter in the unconstrained optimization problem, which is typically assigned a relatively large value to enforce narrow-band signal decomposition. The update formula for the center frequency *ω_L_* is given as follows:

(11) $$\omega_{L}^{n+1}=\frac{\int_{0}^{\infty }\omega {\left|{\hat{u}}_{L}^{n+1}(\omega )\right|}^{2}d\omega }{\int_{0}^{\infty }{\left|{\hat{u}}_{L}^{n+1}(\omega )\right|}^{2}d\omega }$$

The Lagrange multiplier λ can be computed using the dual ascent method, and the corresponding update formula is given by

(12) $${\hat{\lambda }}^{n+1}={\hat{\lambda }}^{n}+\tau \left[\hat{f}(\omega )-\left({\hat{u}}_{L}^{n+1}(\omega )+y(\omega )+\sum_{i=1}^{L-1}u_{i}^{n+1}(\omega )\right)\right]$$

The parameter τ is the update step size, and the Alternating Direction Method of Multipliers (ADMM) is employed to iteratively solve the minimization problem, yielding the expression for y(ω) as

(13) $$y(\omega )=\frac{\alpha_{m}^{2}{\left(\omega -\omega_{L}^{n+1}\right)}^{4}\left(\hat{f}(\omega )-{\hat{u}}_{L}^{n+1}(\omega )-\sum_{i=1}^{L-1}{\hat{u}}_{i}(\omega )+\frac{\hat{\lambda }(\omega )}{2}\right)}{1+\alpha_{m}^{2}{\left(\omega -\omega_{L}^{n+1}\right)}^{4}}-\frac{\sum_{i=1}^{L-1}{\hat{u}}_{i}(\omega )}{1+\alpha_{m}^{2}{\left(\omega -\omega_{L}^{n+1}\right)}^{4}}$$

The SVMD algorithm proceeds as follows:

(1) Input the signal *f*(*t*), and set the internal convergence tolerance *ε*_1_ > 0, external convergence tolerance *ε*_2_ > 0, and additive white noise power δ^2^. Initialize the penalty parameter α and set the number of modes *L* = 0;

(2) Increment L←L+1, and initialize ${\hat{u}}_{L}^{1}$, ${\hat{\lambda }}_{L}^{1}$, and $\omega_{L}^{1}$. Set the iteration index *n* = 0 to start the outer loop;

(3) Repeat for *n*←*n*+1: for all ω > 0, perform the inner loop:

Update ${\hat{u}}_{L}$ according to Equation (10); update $\omega_{L}$ according to Equation (11); update $\hat{\lambda }$ using Equations (12) and (13);

(4) Check the inner loop convergence criterion:

If satisfied $\frac{\|{\hat{u}}_{L}^{n+1}-{\hat{u}}_{L}^{n}{\|}_{2}^{2}}{\|{\hat{u}}_{L}^{n}{\|}_{2}^{2}}<\varepsilon_{1}$, terminate the inner loop;

(5) Check the outer loop convergence. If the decomposition satisfies the predefined condition based on $\frac{\left|\delta^{2}-\frac{1}{T}{\left\|f(t)-\sum_{i=1}^{L}u(t)\right\|}_{2}^{2}\right|}{\delta^{2}}<\varepsilon_{2}$, terminate the algorithm.

## 4. Numerical Simulation

### 4.1. Design of Vehicle-Bridge Interaction (VBI) for Simply Supported Bridges

**Figure 2:**
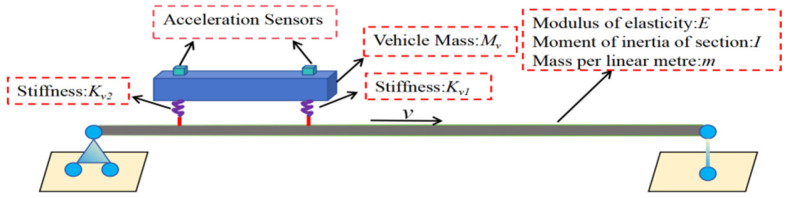
Two-axle test vehicle moving along the bridge.

In the finite element simulation, the total length of the bridge is set to 30 m, divided into 30 elements, each 1 m in length. The number of beam elements has a critical impact on the vehicle–bridge interaction (VBI) response. This is because the damage extent is directly related to the element size used to model the stiffness loss representing damage. It is important to note that, unless otherwise specified, the parameter values remain consistent throughout the subsequent analyses. The time step for analysis is set to 0.001 s. Additionally, the vehicle speed is set to 5 m/s, and the parameters of the vehicle–bridge system are listed in Table 1. The vehicle–bridge interaction (VBI) is implemented using a custom-developed program in Abaqus software. Figure 2 shows the schematic diagram of the two-axle test vehicle moving along the bridge. The theoretical and FEM frequencies of the vehicle–bridge system are provided in Table 2.

**Figure 3:**
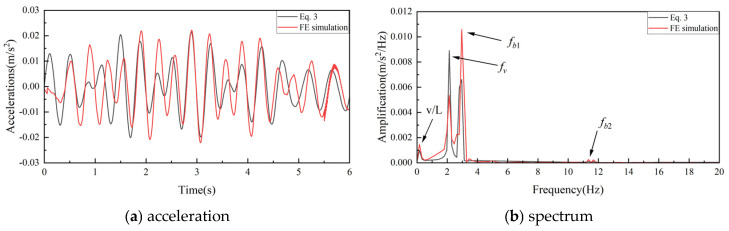
Vehicle acceleration response.

As the two-axle test vehicle crosses the bridge at a speed of v = 5 m/s, the acceleration and spectrum responses for the vehicle are shown in Figure 3. Figure 3 presents the vehicle body acceleration responses and their corresponding Fourier transform results obtained using Equation (3) and the finite element (FE) method. As shown in Figure 3a, a slight deviation exists between the acceleration responses derived from Equation (3) and those from the FE method. This discrepancy can be attributed to the simplifications made during the derivation of Equation (3), where the asymmetric excitation caused by the front and rear axles of the two-axle vehicle entering the bridge sequentially was not considered. Nevertheless, Figure 3b demonstrates that the frequency-domain responses obtained via both methods are largely consistent, thereby confirming the accuracy and validity of the developed finite element model.

A total of 13 damage cases were considered, as shown in Table 3. Under Cases 1–5, it was assumed that the damage was located at the 15th element, with damage levels of 2 percent, 5 percent, 10 percent, 20 percent, and 30 percent, respectively. In Cases 6–8, the damaged elements are located at element 8, element 23, and element 27, respectively, with the same parameters as in Damage Case 5. Case 9 sets up a multi-damage scenario to verify the sensitivity of the proposed metrics to multiple damage locations. In Cases 10 and 11, the vehicle speed was increased from 5 m/s to 8 m/s and 10 m/s to further investigate the effect of vehicle speed. In Cases 12 and 13, we investigated the effect of the number of bridge spans on the damage identification, and arranged the damage cases of two-span and three-span beams.

### 4.2. Extraction of Bridge Frequencies by EMD or SVMD

Frequency is a key parameter that characterizes the dynamic properties of bridges and plays a crucial role in structural health monitoring and assessment. To evaluate the effectiveness of SVMD techniques in extracting bridge frequencies, we applied the method to the vehicle acceleration response, as discussed in Section 4.1.

**Figure 4:**
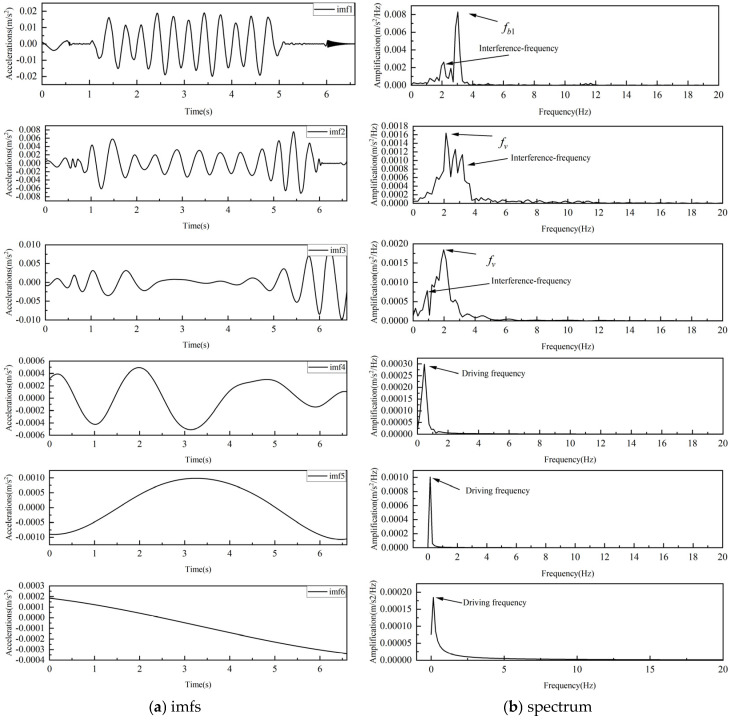
Extraction of frequencies from vehicle acceleration response by EMD.

Figure 4 presents the results of applying the Empirical Mode Decomposition (EMD) method to the vehicle acceleration response. A total of six Intrinsic Mode Functions (IMFs) are shown. Figure 4a displays the time-domain IMFs, while Figure 4b illustrates their corresponding frequency-domain representations. It can be observed that the EMD only identifies the first natural frequency of the bridge. This is attributed to the relatively low energy contribution of the second mode in the vehicle response. Additionally, the decomposition results exhibit mode mixing among the IMFs, and the frequency peaks of IMF4 to IMF6 correspond to the driving frequencies.

**Figure 5:**
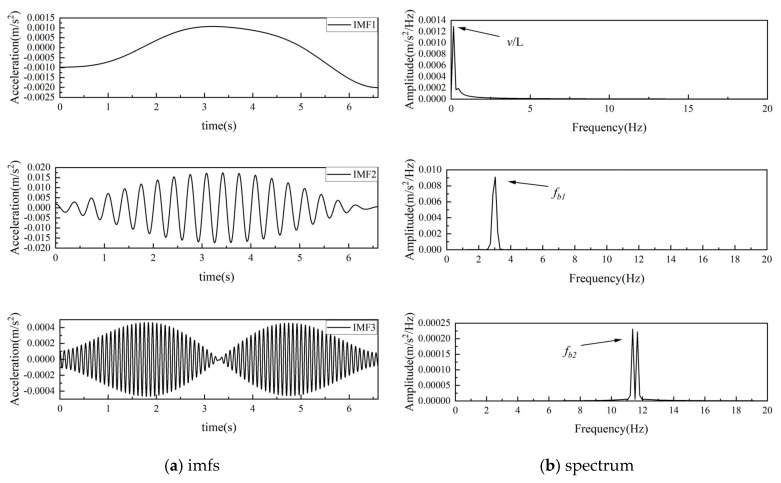
Extraction of frequencies from vehicle acceleration response by SVMD.

Figure 5a shows the three IMFs obtained through SVMD, with their corresponding frequency domain representation presented in Figure 5b. As shown in the chart, SVMD effectively identified the driving frequency and the first two bridge frequencies without modal aliasing among the IMFs and providing smooth and refined IMFs.

### 4.3. Damage Identification Based on SVMD

Previously, the damage indicators constructed by VSM-based bridge damage identification are easily interfered by measurement noise and road roughness. This study proposes a new VSM-based bridge damage identification method for small and medium-span bridges, and the damage indicators are established based on the vehicle acceleration response by extracting the IMF corresponding to the driving frequency components using the SVMD technique.

The steps of damage identification in this study are as follows:

Step1: Record vertical acceleration response of the two-axle vehicle;

Step2: Using SMVD to process vertical acceleration, FFT is performed to find the IMF corresponding to the driving frequency;

Step3: Extraction of driving frequency IMF*_u_* (unknown state) and IMF*_s_* (initial state);

Step4: Damage indicator = IMF*_u_* − IMF*_s_*.

It is worth noting that the difference in IMFs between the unknown state and the initial state (not necessarily undamaged state) is discussed here because structural health monitoring is a long-term task, and we are more concerned with the changes in the structural state during the monitoring period. When used in practical engineering, it is not necessary to predict the initial state of the structure, but only need to compare the difference in the distribution of the acceleration time-range signals at the driving frequency IMF collected by the vehicles crossing the bridge twice to judge whether the structural state has changed in the meantime.

### 4.4. Damage Severity Identification

**Figure 6:**
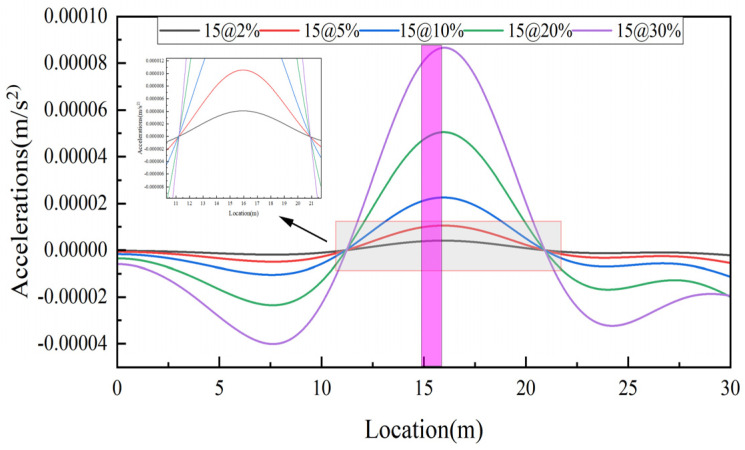
Identification results at different levels of damage.

Keep bridge and vehicle parameters unchanged, the damage of 2%, 5%, 10%, 20%, and 30% is set in the 15th element, corresponding to Cases 1–5. The damage identification results are shown in Figure 6. From the figure, it can be seen that the damage becomes more obvious as the damage degree increases. Therefore, a preliminary judgement of the damage degree can be made by the size of the peak at the damage location. In addition, for the small damage in Case 1 and Case 2, the damage indicator can also clearly and accurately identify the damage location, indicating that the damage indicator has a high sensitivity to damage.

### 4.5. Identification at Different Damage Locations

**Figure 7:**
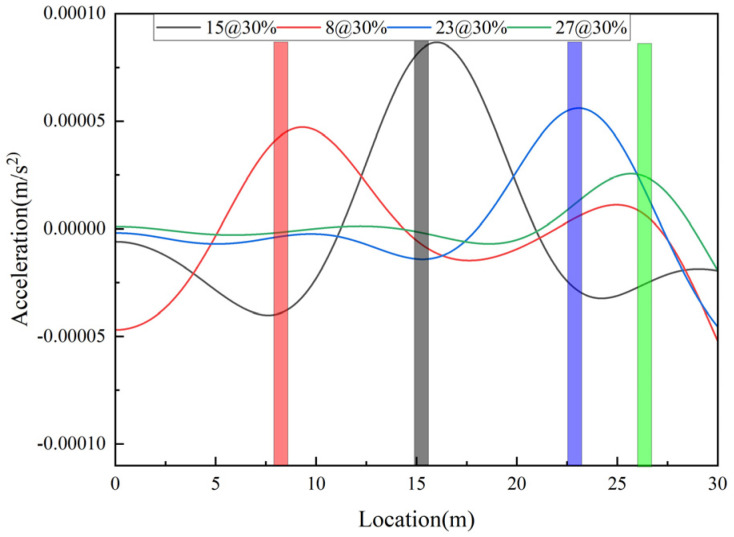
Identification at different damage locations.

Keep bridge and vehicle parameters and the damage level unchanged, set the damage at the 8th, 23rd, and 27th elements, respectively, and the damage identification results are shown in Figure 7. As can be seen from the figure, the damage indicators can be accurately identified for different damage locations. However, for different damage locations, the size of the peak at the damage location varies. In general, the closer to the span center, the larger the peak at the damage location; the closer to the end point, the smaller the peak at the damage location.

### 4.6. Multi-Damage Identification

**Figure 8:**
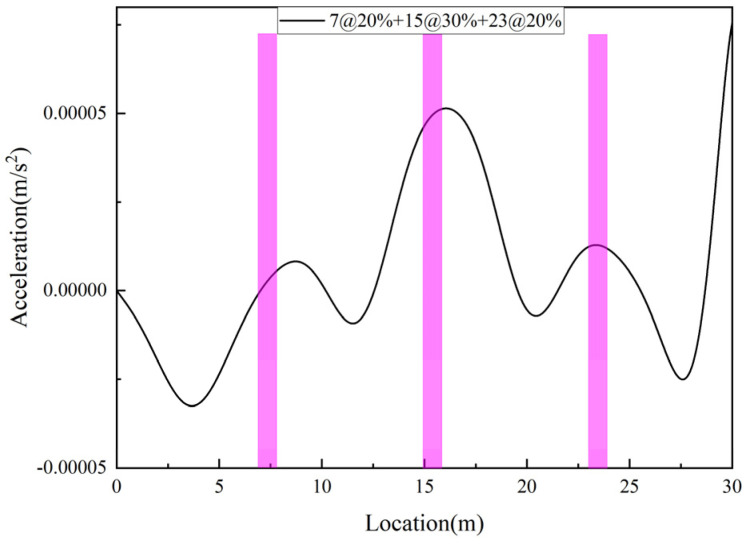
Multi-damage location identification.

Keep bridge and vehicle parameters unchanged, and set 20%, 30%, and 20% damage in elements 7, 15, and 23, respectively, corresponding to Case 9. The damage identification results are shown in Figure 8. From the figure, it can be seen that the peaks of the damage locations basically coincide with the preset damage locations, indicating that the damage indicator can accurately identify each damage.

## 5. Parameter Discussion

Considering the different speeds and bridge spans in the actual damage detection process, the damage identification effect of the damage indicators will be different and the vehicle–bridge system will also be interfered by the roughness of the road surface. Therefore, this section will discuss the above parameters and influencing factors to analyze the impact of each parameter on the damage identification effect.

### 5.1. Effect of Vehicle Speed

**Figure 9:**
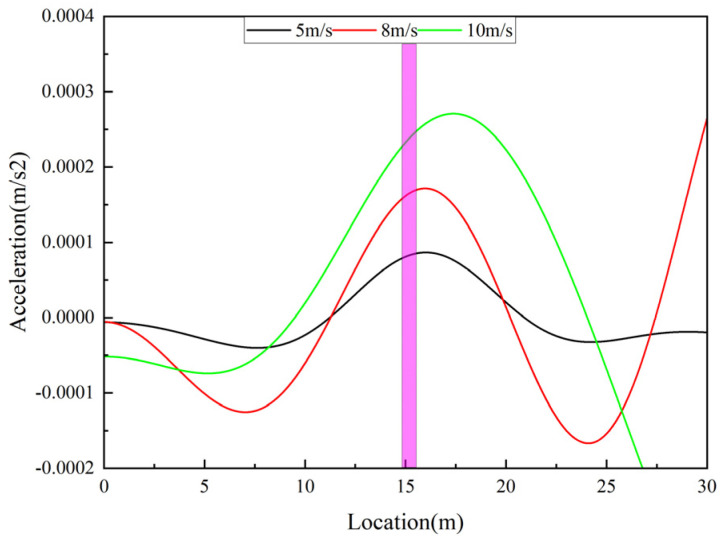
Identification results at different vehicle speed.

In the previous methods for bridge damage identification using VSM, the vehicle is required to keep a low speed to ensure the spatial resolution, thus limiting the application of the method. In this section, the vehicle speed is discussed, keeping the bridge and vehicle parameters, bridge damage location, and damage degree unchanged, and the damage identification results at different vehicle speeds are shown in Figure 9. From the figure, it can be seen that the peak value at the damage location keeps increasing with the increase of vehicle speed. As the vehicle speed increases, the amplitude of the vehicle response also rises, which enhances the mutual excitation between the vehicle and the bridge, thereby amplifying the changes induced by structural damage. On one hand, the increase in driving speed elevates the driving frequency, facilitating its extraction. On the other hand, higher speeds shorten the data acquisition duration, reducing the amount of collected data and potentially leading to localization errors in damage identification. Moreover, excessive speeds may exacerbate the interference caused by road surface irregularities. Therefore, to improve the accuracy and reliability of damage identification, it is recommended that the vehicle speed be kept below 8 m/s in practical applications.

### 5.2. Effect of Pavement Roughness

**Figure 10:**
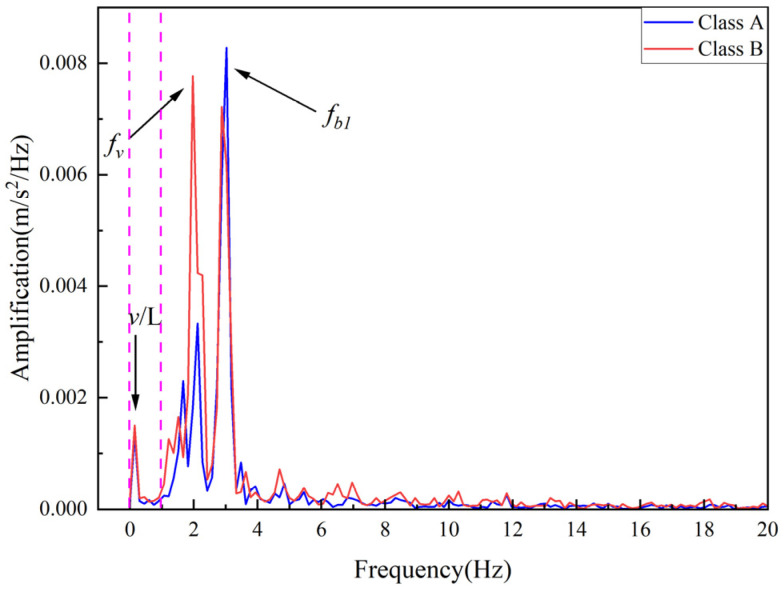
Amplitude–Frequency spectrum under Class A and Class B pavement roughness.

**Figure 11:**
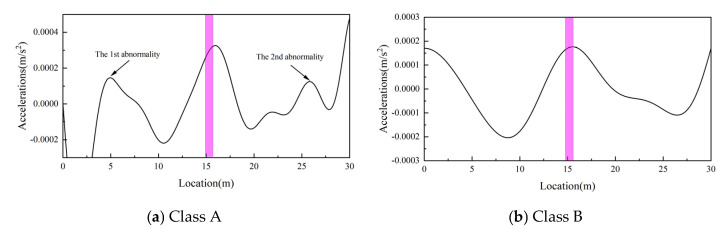
Class A and Class B pavement roughness identification results.

In reality, bridge surfaces have a certain roughness, which amplifies the excitation effect on vehicles and introduces interference frequencies. In order to study the effect of pavement roughness on the damage identification method, in this study, we considered the addition of Class A pavement roughness and Class B pavement roughness for Case 4, respectively. The spectrum of the vehicle acceleration response for this condition is shown in Figure 10, where the pavement roughness spectrum is generated according to the method described in reference [38]. After considering the road roughness, the entire frequency domain range contains the frequency components of the road roughness, so band-pass filtering is first used to remove the interfering frequencies. Considering that only the IMF corresponding to the driving frequency is concerned, and the vehicle frequency is 2.12 Hz, the band-pass filtering range is set to 0.01–1 Hz. The filtering is followed by SVMD decomposition, and then the damage metrics are calculated. The damage identification results are shown in Figure 11. According to Figure 11, the damage indicator can still accurately identify the damage location under the consideration of Class A pavement and Class B pavement. It is worth noting that the sequential entry and exit of the front and rear axles of a two-axle vehicle can influence the accuracy of damage identification by affecting the selection of the filtering range around the driving frequency. A wider filtering range tends to enhance the visibility of damage-related features, thereby improving localization accuracy. However, an excessively broad range may introduce additional interference components, compromising the robustness of the results. Therefore, an appropriate and adaptive selection of the filtering range is essential to achieve optimal damage detection performance.

### 5.3. Effect of Number of Bridge Spans

**Figure 12:**
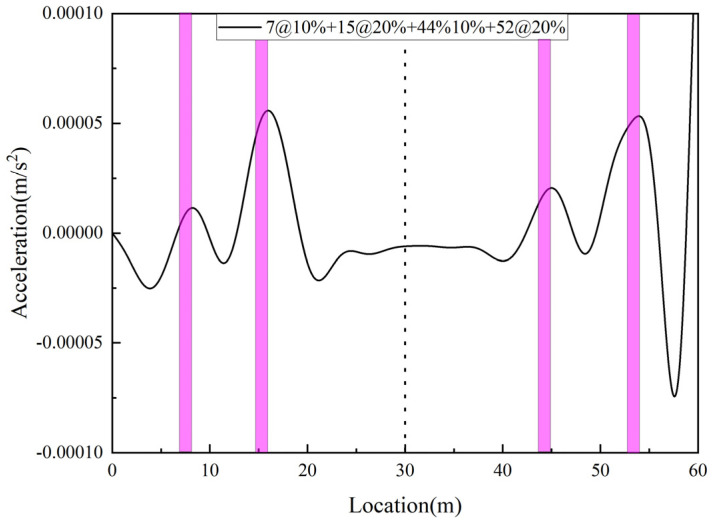
Two-span bridge damage identification result.

**Figure 13:**
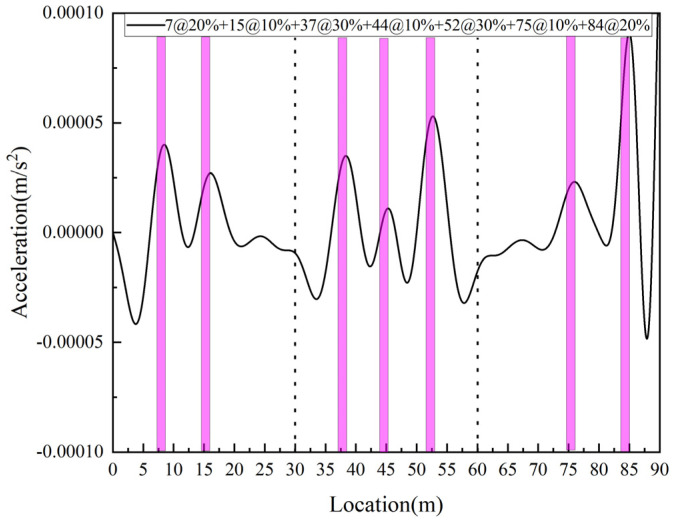
Three-span bridge damage identification result.

In the above analysis, only the single span simply supported beam was considered. Through the above discussion, it is useful to use this damage index for damage identification of simply supported beams, while in reality, most of the bridges are continuous. Therefore, in this section, two-span and three-span beams will be studied to verify the validity of the proposed damage index for continuous bridges, assuming that the length of each span is equal to the length of a single span. A two-span bridge is considered in Case 12, where elements #7 and #15 of the first span are set at 10% and 20% damage, respectively, and elements #44 and #52 of the second span are set at 10% and 20% damage, respectively. In Case 13, a three-span bridge is considered, in which elements #7 and #15 of the first span are set with 20% and 10% damage, respectively, elements #37, #44, and #52 of the second span are set with 30%, 10%, and 30% damage, respectively, and elements # 75 and #84 of the third span are set with 10% and 20% damage, respectively. The damage identification results obtained are shown in Figure 12 and Figure 13, respectively. From the figure, it can be seen that both two-span and three-span beams show peaks at the damage location, and the proposed damage identification indexes are able to accurately identify the location of the damage.

## 6. Experimental Study

### 6.1. Description of the Laboratory Test

**Figure 14:**
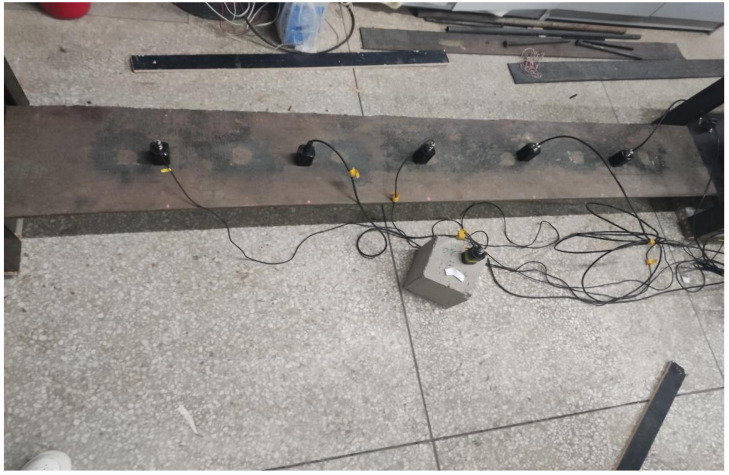
Bridge model.

The bridge model, illustrated in Figure 14, comprises a main girder constructed from steel plates. The girder measures 2000 mm in length, 300 mm in width, and 15 mm in thickness, with a total mass of approximately 68 kg. The bridge’s support system consists of a “top block + roller + bottom block” configuration. One roller is welded to the top block, creating a fixed connection that permits only rotational movement, thereby acting as a fixed hinge support. The other roller is unattached to either the top or bottom block, enabling both rotational movement and slight longitudinal displacement, which simulates a simple hinge support.

**Figure 15:**
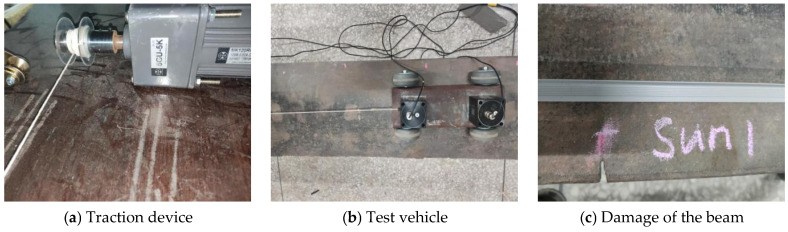
Vehicle model and traction system.

The two-axle test vehicle is powered by a variable frequency motor, as shown in Figure 15a. It is secured to the motor’s roller drum with a nylon rope, enabling the vehicle to maintain a constant speed. The vehicle model is illustrated in Figure 15b, featuring a main body constructed from steel plates measuring 300 mm × 200 mm × 5 mm, with a total weight of 2.4 kg. Each wheel is fitted with rubber tires, and the performance and spring stiffness of the wheels are generally consistent. An accelerometer is installed at the center of both the front and rear axles. Measurement data is collected using a DH5922 data acquisition device (Jiangsu Donghua Testing Technology Co., Ltd., Jingjiang, China), which has a sampling frequency of 1000 Hz. The accelerometers have a frequency range from 0.25 Hz to 100 Hz. A total of two damage cases were set up for the experimental beam. There were two damage locations at 0.6 m and 1.0 m of the beam, which were achieved by cutting 20 mm depth at the edge of the beam as shown in Figure 15c.

### 6.2. Bridge Direct Test Results

**Figure 16:**
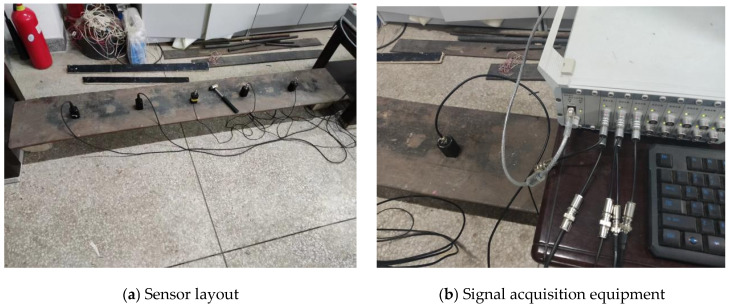
Direct measurement.

To verify the accuracy of the subsequent vehicle scanning method results, the bridge structure’s frequencies were first measured using conventional direct measurement techniques. A total of five piezoelectric velocity sensors were distributed across the span and a sampling frequency of 1000 Hz. The mass and volume of each sensor are negligible. A force hammer was struck at the position of 1/3L, and the response and excitation were recorded using the DHDAS dynamic signal acquisition and analysis system, applying the principle of frequency response functions to identify the bridge’s frequencies. Figure 16 illustrates the arrangement of the sensors and signal acquisition equipment. By analyzing the signals collected from multiple force hammer strikes, the first two bridge frequencies were ultimately determined. The first two frequencies of the model bridge were found to be 10.37 Hz and 40.64 Hz.

### 6.3. VSM Test Results

**Figure 17:**
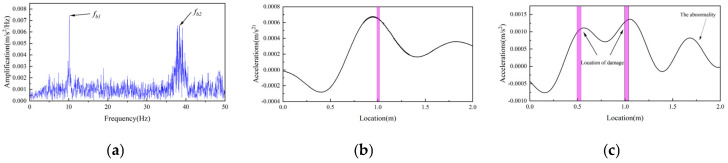
Results of damage identification. (**a**) Vehicle acceleration amplitude–frequency. (**b**) Single damage identification. (**c**) Multi-damage identification.

In order to verify the effectiveness of the proposed bridge damage identification method, a two-axis test vehicle was driven over the steel girder at a speed of 0.1 m/s. The spectrum obtained from the acceleration signal of the vehicle is shown in Figure 17a, from which it can be seen that the first-order frequency of the bridge can be clearly recognized, with a value of 10.167 Hz, which is only 2.36% different from the direct test result. The results of damage identification are shown in Figure 17b,c. From Figure 17b, it can be seen that the location of the damage can be identified based on the IMF difference; however, there is a deviation in the identified damage location. The reason for this is analyzed to be due to local vibration of the vehicle and measurement noise interference. As shown in Figure 17c, the two damage locations identified by the damage indicator align well with the actual damage positions. However, an additional spurious peak is observed. This artifact is attributed to a bounce (lift-off) of the experimental vehicle as it exited the bridge, which induced a transient disturbance and resulted in a pronounced false peak in the response signal. In summary, the damage indicators proposed in this study can basically identify the damage identification; however, due to the serious influence of local vibration of the vehicle and measurement noise, the identification accuracy is low.

## 7. Concluding Remarks

In this study, a theoretical study of damage identification of bridges is carried out based on the acceleration response of a biaxial test vehicle. Firstly, the theoretical solution of the dynamic response of the biaxial test vehicle is derived. Subsequently, the driving frequency-dependent IMF is separated from the vehicle acceleration response using the SVMD technique for damage identification. The above calculation results were verified by finite element analysis and experimental tests to ensure the accuracy and reliability of the proposed method. Based on the theory and analysis, the following conclusions are drawn:

(1) SVMD decomposition provides a fine method for processing complex signals. It can effectively avoid modal aliasing, thus obtaining smooth and clear intrinsic modal functions (IMFs).
(2) The proposed damage index can successfully identify the location of single and multiple damages of a bridge.
(3) The proposed method can effectively identify the damage identification of bridges even under the influence of pavement roughness.
(4) From the experimental results, the damage identification accuracy is low due to the more serious interference from the vehicle’s own local vibration and measurement noise.

It is important to note that in bridge structures, modal parameters exhibit spatial distribution characteristics. When damage occurs near the mid-span, it tends to have a more significant impact on the system’s dynamic response, often leading to amplified peak values. In contrast, damage near the supports results in a comparatively weaker effect. Consequently, the amplitude of the response peaks is influenced not only by the severity of the damage but also by its spatial location within the structure. Identical levels of damage at different positions may produce distinct response intensities. In future studies, we aim to address this positional dependency by establishing a mapping relationship between the damage index and its corresponding location using finite element models or experimental data. Alternatively, position-based normalization of peak values will be considered to mitigate the influence of spatial variability, thereby enhancing the robustness and reliability of the proposed damage indicators. Meanwhile, to enhance the intelligence and automation of the proposed approach, future research will focus on decomposing the acquired time-domain signals using SVMD, followed by the application of Continuous Wavelet Transform (CWT) to generate time-frequency representations. These representations will then be integrated with deep learning techniques for improved accuracy and efficiency in damage identification.

In the experiment, structural damage was simulated by introducing a 20 mm deep notch at a critical location on the beam, aiming to create a localized stiffness reduction that alters the bridge’s modal properties. While this approach provides a controlled and reproducible damage scenario for method validation, we acknowledge that it is a simplification compared to real-world bridge deterioration such as fatigue cracking, corrosion-induced section loss, or concrete delamination. Real damage tends to be progressive, spatially distributed, and sometimes nonlinear in nature, which may result in more complex or subtle changes in dynamic response, including nonstationary or mode-coupling behaviors. Moreover, early-stage damage may not cause sufficiently noticeable shifts in modal parameters, posing challenges for detection methods based solely on global modal variation. Therefore, the notch-based damage scenario used in this work primarily serves to validate the proposed method under idealized stiffness loss conditions. In future studies, we plan to incorporate more realistic damage forms, such as fatigue crack propagation, corrosion simulations, or material degradation, to evaluate the robustness and sensitivity of the method under more complex and practical damage scenarios.

In light of the observed damage localization deviations and spurious peak interferences in the experimental results, our future work will incorporate more advanced denoising techniques, such as wavelet thresholding combined with Variational Mode Decomposition (VMD) or Successive Variational Mode Decomposition (SVMD), to more effectively suppress irrelevant signal components. Additionally, post-processing strategies based on statistical analysis or machine learning methods—such as Principal Component Analysis (PCA) and Support Vector Machines (SVMs)—will be introduced to distinguish genuine modal shifts from those caused by environmental variations or random noise.

Although this study focuses on simply supported and continuous beam bridges, we argue that the proposed method demonstrates strong scalability. This is primarily because the approach centers on the identification of vehicle-induced frequencies, which are universally present across all types of bridges—including simply supported, continuous, and long-span bridges. As such, the method is inherently geometry-independent and can be readily extended to longer or more complex structures. Moreover, the SVMD algorithm offers adaptive decomposition of complex signals with overlapping modes. Long-span bridges often exhibit closely spaced low-frequency modes. Compared with traditional techniques such as EMD or conventional filtering, SVMD achieves higher modal separation resolution, making it particularly suitable for large-scale modal identification in complex bridge systems.

The proposed bridge damage identification approach, which combines vehicle response signals with successive variational mode decomposition (SVMD), has demonstrated strong sensitivity and localization capability under various scenarios. However, we acknowledge certain limitations regarding its applicability to different damage types and orientations. Specifically, since the vehicle-mounted sensors primarily capture vertical acceleration responses, the method exhibits higher sensitivity to damage types such as cracking at the bottom of the beam. In contrast, its effectiveness may be limited for shear-type damage, transverse cracks, or minor damage that does not significantly alter the global dynamic characteristics of the bridge. To enhance the generalizability and sensitivity of the proposed method, future work will explore the integration of multi-pass vehicle data fusion, localized response feature extraction, and finite element model-assisted diagnostics. These enhancements are expected to improve the method’s adaptability to various types and orientations of structural damage.

## Figures and Tables

**Table 1:** Vehicle and bridge parameters.

Vehicle	Mass *M_v_* (kg)	2500
	Front Axle Stiffness *K_v_*_1_ (kN/m)	230
	Rear Axle Stiffness *K_v_*_2_ (kN/m)	230
	Moment of Inertia Jv (kg·m^2^)	2300
	Wheelbase *d* (m)	3
	Distance from Front Axle to Center of Gravity *d*_1_ (m)	1.5
	Distance from Rear Axle to Center of Gravity *d*_2_ (m)	1.5
	Vehicle Speed *v* (m/s)	5
Bridge	Span Length *L* (m)	30
	Elastic Modulus *E* (GPa)	27.5
	Moment of Inertia of Cross-Section *I* (m^4^)	0.2
	Mass per Unit Length *m* (kg/m)	2000

**Table 2:** Theoretical and FEM frequencies of the vehicle–bridge system.

Bridge/Vehicle Mode	First Bridge (*f_b_*_1_)	Second Bridge (*f_b_*_2_)	Vehicle Vertical (*f_v_*)
Theoretical Frequency (Hz)	2.89	11.58	2.16
FEM Frequency (Hz)	3.03	11.51	2.12

**Table 3:** Damage cases.

Case Number	Vehicle Mass (kg)	Vehicle Speed (m/s)	Damage Location (Element Number)	Degree of Damage (%)
1	2500	5	15	2
2	2500	5	15	5
3	2500	5	15	10
4	2500	5	15	20
5	2500	5	15	30
6	2500	5	8	30
7	2500	5	23	30
8	2500	5	27	30
9	2500	5	7, 15, 23	20, 30, 20
10	2500	8	15	30
11	2500	10	15	30
12	2500	5	7, 15, 44, 52	10, 20, 10, 20
13	2500	5	7, 15, 37, 44, 52, 75, 84	20, 10, 30, 10, 30, 10, 20

## Data Availability

All data, models, or codes that support the findings of this study are available upon reasonable request.
